# Menopausal symptoms and work: a narrative review of women’s experiences in casual, informal, or precarious jobs

**DOI:** 10.1016/j.maturitas.2021.05.007

**Published:** 2021-05-31

**Authors:** Heather Yoeli, Jane Macnaughton, Sarah McLusky

**Affiliations:** Institute for Medical Humanities & Department of Anthropology, Durham University, UK

**Keywords:** menopause, employment, work, discrimination, poverty, COVID-19

## Abstract

Governments, employers, and trade unions are increasingly developing “menopause at work” policies for female staff. Many of the world’s most marginalised women work, however, in more informal or insecure jobs, beyond the scope of such employment protections. This narrative review focuses upon the health impact of such casual work upon menopausal women, and specifically upon the menopausal symptoms they experience. Casual work, even in less-then-ideal conditions, is not inherently detrimental to the wellbeing of menopausal women; for many, work helps manage the social and emotional challenges of the menopause transition. Whereas women in higher status work tend to regard vasomotor symptoms as their main physical symptom, women in casual work report musculoskeletal pain as more problematic. Menopausal women in casual work describe high levels of anxiety, though tend to attribute this not to their work as much as their broader life stresses of lifelong poverty and ill-health, increasing caring responsibilities, and the intersectionally gendered ageism of the social gaze. Health and wellbeing at menopause is determined less by current working conditions than by the early life experiences (adverse childhood experiences, poor educational opportunities) predisposing women to poverty and casual work in adulthood. Approaches to supporting menopausal women in casual work must therefore also address the lifelong structural and systemic inequalities such women will have faced. In the era of COVID-19, with its devastating economic, social and health effects upon women and vulnerable groups, menopausal women in casual work are likely to face increased marginalisation and stress. Further research is need.

## 1 Introduction

Recent UK studies [1–5] and reviews of the global literature [6–8] have tended to regard employment and work as synonymous with one another. Across the world, however, many menopausal women^1^ are not formally employed but nevertheless undertake ‘informal’, ‘sessional’, ‘precarious’ or ‘casual’ work, and in the so-called ‘grey’ economy, beyond the scope of taxation and employment protections [10]. For the most intersectionally marginalised menopausal women, work is not necessarily employment, and work that is not employment is often the most problematic form of work.

Whereas some casual workers operate relatively autonomously and can organise their workloads independently [11], others are closely managed or exploited by managers acting beyond the scope of employment legislation, and many are left unsure as to when they have work and how much and when they might be paid [12]. Whereas casual work may benefit the wellbeing of young people [13], it is generally regarded as detrimental to the health of adult workers with social and financial responsibilities, and as particularly detrimental to health of adult female workers [14]. Therefore, this literature review focuses upon the health impact of casual work upon menopausal women, and specifically upon the nature and determinants of menopausal symptoms experienced by women in casual work.

## 2 Background

Historically, women from socioeconomically marginalised groups have often worked ‘cash in hand’ from home in the so-called ‘grey’ economies, doing for example piecework sewing, cake-decorating and network marketing [15]. Beyond the home, women have long worked in ‘informal’, ‘sessional’ or ‘casual’ roles, for example as babysitters, agricultural pickers, and home care workers. In recent years, however, the growth of zero-hours contracts and the so-called ‘gig economy’ operating beyond conventional structures and safeguards of employment procedures legislation and policy [10] has meant that casual work exists throughout the traditionally ‘working-class’ and ‘female-dominated’ cleaning, retail, catering and care industries [14].

Women have always had a complicated relationship with the concept of work [8, 16], particularly when facing competing obligations and responsibilities from their personal lives. For many women, the menopause is a time when the pressure of these competing duties intensifies. A lingering social archetype of the menopausal woman as a calm, wise and dependable carer [9, 17] often combines with the intersectionally gendered ageism that undermines the credibility and self-confidence of menopausal women at work [4, 5, 8]. Menopausal women are often expected to set aside their own career aspirations and financial wellbeing to care for adult children, grandchildren, for elderly parents or grandparents or for other sick or disabled relatives and friends [7, 8], and even though they may still have their own children at home and may be dealing with their own health challenges [1, 8]. For some menopausal women, casual work or the grey economies may provide their only means of earning money whilst working in or near the home, or of working hours which accommodate their caring or health needs. Often, however, the ‘flexibility’ of such roles transpires as largely to the benefit of their company [18]; given that casual work falls beyond the reach employment legislation and protections, it can often be insecure, underpaid, hazardous or exploitative. Menopausal women in casual jobs may therefore to be working this way by necessity as well as choice, unable to secure formal employment due either to a lack of local opportunities, or to gendered ageism and other forms of disadvantage, or to their own lack of qualifications and personal work history [10].

Since early 2020, the economic impact of the COVID-19 pandemic has been particularly intense for women in causal or precarious work in the retail, travel, catering, and hospitality, sectors which have been subject to substantial job losses worldwide [19]. Together with rising levels of social inequality and gender-based violence, COVID-19 has proved hugely detrimental to the rights, wellbeing, and safety of women, undermining many years of progress towards gender equality [20]. It is likely that the most vulnerable menopausal women, who were already experiencing high levels of disadvantage and marginalisation at work, will have been particularly adversely affected.

## 3 Methods

We searched the eight academic databases found by previous reviews [6–8] to yield the most relevant results, adding Google Scholar as another widely-used resource. To establish findings of contemporary relevance, we restricted all searches to 1995 or later.

Our search protocol (see Figure 1) included the keyword terms used by previous reviews [6–8]. Like Jack et al. [6], we discovered that ‘work’ was a slightly ambiguous keyword, and thereby included their term ‘employ*’ as a synonym, even though our study sought research into casual and grey economy work rather than employment. Initial searches used the keywords ‘the change’ and ‘menopause transition’ as popular UK synonyms for the menopause [5, 8, 9]. However, we found the multiple meanings of ‘change’ and ‘transition’ yielded many irrelevant results. As our search protocol developed (see Figure 1), we found casual or exploitative working practices amongst menopausal women to be particularly associated with migration and poverty, so included these and their synonyms as a keyword term.

These searches produced 108 shortlisted primary research studies related to the menopause and work. We surveyed their reference lists of each in search of further potentially relevant literature, adding 3 studies as a result. We read and appraised each of the 111 shortlisted.

We included all qualitative studies which provided sufficient demographic information to suggest all or most menopausal women participants were in casual work (Group I, *n*=3 [21–23]).

Amongst larger-scale population-based cohort studies of menopause and work, we included those which disaggregated their data to distinguish their findings on casual work and from other forms of employment (Group II, *n*=5 [24–28]).

The smaller-scale workplace-based and community-based quantitative studies tended to offer little contextual detail about either their participants or the workplace structures or conventions. Therefore, we found it more difficult to ascertain whether we should regard these participants as in casual work or employed. Acknowledging that the boundary between casual work and employment is ultimately a socioeconomic construct, we included all surveys which disaggregated the experiences of menopausal women in insecure, manual, low-paid, unskilled, hazardous or exploitative work from those in other roles, even when we could not be certain that participants had not been employed in their roles (Group III, *n*=5 [29–33]).

Included studies are classified and detailed in Figure 2. These were analysed using the MOOSE protocol [34]. In presenting the findings we draw out how women reported and assessed symptoms of the menopause, and how they addressed them to elicit any distinctions between the experiences of women in formal and casual work.

## 4 Findings

### 4.1 Musculoskeletal symptoms

For women in casual work, musculoskeletal symptoms of joint and muscle stiffness, aches, and pains, particularly in the legs, back, shoulders and neck, were the commonest and worst symptom of the menopause [22, 23]. Within cohorts of employed women by contrast, hot flushes were slightly [33] or significantly [31] more prevalent.

Menopausal women in casual work with a strong manual or menial component reported these musculoskeletal symptoms as having a markedly detrimental effect on their work performance [22], in many cases causing them to leave their jobs in order to seek less physically-demanding work elsewhere [23]. These women found musculoskeletal pain particularly difficult to manage, feeling as though nothing they could do would relieve their symptoms [21, 23].

### 4.2 Psychological symptoms

Within the qualitative literature, lifelong poverty seemed to provide precariously employed women with experiences of and resilience to emotional stress and mental ill-health which predated their menopause [21, 22]. These women described feeling more able to manage the psychological aspects of the menopause than their musculoskeletal symptoms [21, 23]. Though nevertheless affected by anxiety [21] or feeling tense and being touchy or irritable [23], women asserted that they were able to manage emotions by working harder [23], especially when their income, however precarious, relieved some financial worries and provided some economic independence [22].

This finding concurs with the workplace-based surveys comparing ‘working’ and ‘housewife’ status; irrespective of the nature of their job, women in work found the psychological symptoms of the menopause less problematic than housewives did [29, 31, 33]. Beyond empowerment, work can provide women with opportunities to overcome taboos around menstruation, emotions and ageing to talk and learn about the menopause and to seek medical help for symptoms [31–33]. Nevertheless, menopausal women in lower-paid and more manual jobs experience significantly more psychological symptoms than women in higher status ‘white collar’ jobs [31, 35].

Both qualitative and quantitative studies highlight the importance to emotional wellbeing of menopausal women’s self-image in response to the social gaze, stereotypes and expectations associated with ageing [22, 23, 32]. For many, anxiety was not only a symptom of the menopause but a response to the mounting social pressures placed upon them [21, 23].

### 4.3 Determinants of menopausal symptoms

Within the qualitative studies, menopausal women in casual roles described work as only one part of their daily lives, and as less significant in determining their wellbeing than their social, family, or personal circumstances [21–23]. As such, the women attributed their menopausal symptoms not to their work but instead to the life circumstances which had led them into casual work or the grey economy, described in terms of gendered disadvantage [21–23], poverty [21, 22] and intersectional marginalisation [21, 23].

Within the surveys investigating the socioeconomic determinants of menopausal experience, current working circumstances or conditions consistently showed little if any impact upon age at menopause [24–27], nor quality of life at menopause [28]. Instead, educational history and early childhood adversity were established as the main determinants of menopause experience by European, North American and East Asian studies [24, 25, 27, 28, 31]. In studies undertaken in settings as diverse as the UK [25], France [24], Canada [21], and Turkey [33], working conditions appeared significant only to the extent that they reflect or are determined by a woman’s education or earlier life experience.

## 5 Discussion

### 5.1 Casual work

This review has found that casual work, even in less-then-ideal conditions, is not unambiguously detrimental to the wellbeing of menopausal women. As wider studies concur, any work may be preferable to unemployment [36]. Nevertheless, women in casual work appear more frequently and more severely affected by the musculoskeletal symptoms of the menopause than women more securely employed in jobs with comparable physical demands. This review has found that while menopausal women in casual work may experience similar levels of anxiety and other psychological symptoms to women employed in similarly low-paid and low status jobs; women in casual work seem largely to cope with their psychological symptoms more confidently and more effectively than with their musculoskeletal symptoms.

### 5.2 Symptoms

Few studies have focused upon musculoskeletal symptoms of the menopause at work [37, 38]. Whereas all three previous reviews have discussed the prevalence of psychological symptoms, only one [7] makes mention of musculoskeletal symptoms, even though it found muscle and joint pains to be only incrementally less of a problem than hot flushes [1]. This underrepresentation of musculoskeletal symptoms illustrates the widely-acknowledged lack of research into the physical challenges facing menopausal women in manual work [3, 8]. Care-home employees in physically-demanding yet secure employment reported musculoskeletal problems impaired their working abilities less than the psychological symptoms of the menopause [35]. When compared to employees in more formalised or stable manual work, women in casual work may suffer from musculoskeletal symptoms in particularly severe and disabling ways. However, none of the studies reviewed sought to link specific participant symptoms to particular aspects of their workplaces or working tasks, and all of the studies were published before the 2020 onset of the Covid-19 pandemic led to a rapid increase in home-based working. It is therefore important to emphasise that, by undertaking a narrative review rather than a systematic review or realistic synthesis, we make no attempt to posit any causal mechanisms claiming to explain how casual work might cause musculoskeletal symptoms. More clinically-focused empirical research would be needed to establish clearer understandings of menopause-specific work-related musculoskeletal difficulties.

Previous reviews have emphasised how employed menopausal women frequently struggle to cope with psychological symptoms at work [6–8], and menopausal women employed in professional or clerical positions within large organisations list their problems of concentration, memory and confidence as their foremost workplace challenges [1]. This review, by contrast, has found that menopausal women in casual work are apparently more psychologically resilient, implying that the seemingly most marginalised menopausal women might cope better than more advantaged and employed women. This challenges popular and arguable paternalistic assumptions around menopausal women as in need of the care or help from policy and legislation [9, 17]. Concepts and models of psychological resilience to menopausal difficulties are emerging as explanations for the epidemiology of symptoms [30]. A more asset-based approach to menopause and work research might inform which women cope best with which symptoms and why.

Within menopause and work research and policy, findings around vasomotor symptoms dominate many studies [3, 39]. Certainly, menopausal women find explaining and managing hot flushes at work a uniquely awkward and embarrassing task, even when other symptoms can be more disruptive [2, 4]. However, feminist perspectives critique this disproportionate consideration given to vasomotor symptoms as a manifestation of the intersectional stigmatisation of the older female body, which society has long sought to normalise or to control [16]. Irrespective, then, of how women manage their menopausal symptoms, hot flushes are those that male managers and colleagues find the most difficult to cope with [5]. Musculoskeletal symptoms, by contrast, can be experienced by men as well as women and may therefore be less embarrassing for managers and colleagues.

### 5.3 Determinants

Workplace surveys undertaken amongst menopausal women in ‘white-collar’ or professional roles found high levels of work stress and low levels of job control significantly to exacerbate menopausal symptoms [3, 40]. From this, as well as from the more general employment wellbeing literature [12], it might have been anticipated that this review would find menopausal women in casual work to experience symptoms directly related to this stress and lack of control. Instead, this review found that women in casual work attributed their menopausal symptoms and their difficulties in managing them not to their inadequate or unfair working conditions, but to their broader life circumstances [21–23]. Amongst the multiple everyday challenges these women navigated, work was not necessarily a major part of life, and therefore not necessarily a major stress factor [21, 22, 30].

Across both population-wide and workplace-based surveys of the menopause and work, early childhood adversity is shown as the greatest predictor of menopausal symptoms [26, 28, 41] whereas education [31, 42] is the strongest preventative factor. Similarly, adverse childhood experiences have been shown, independent of education, to determine patterns of employment and work throughout adulthood [43]. Therefore, the association between casual work and a difficult menopause appears to be mediated by the common factors of poor education and early childhood adversity. Menopausal women in casual work may therefore experience the symptoms they do for the same reasons that they are working in casual roles or the grey economy rather than secure employment: because they were raised in poverty and disadvantage, and because they have had few educational opportunities. Therefore, while the precarity, low pay, exploitation, and lack of workplace protections prevalent within the casual sector and grey economy are undoubtedly detrimental to the overall wellbeing of workers [14, 18], casual work cannot be claimed directly to cause or singularly to worsen the symptoms of menopausal women. Health promotion initiatives seeking to improve the wellbeing of menopausal women in casual work [21] are dealing not only with the current working conditions and lifestyles of participants, but with the cumulative legacy of life-long and intersectional adversity and disadvantage.

### 5.4 Impacts of the COVID-19 Pandemic

Research has begun to explore the mental health and broader wellbeing consequences of the stress of care work during the COVID-19 pandemic [41]. However, none has yet considered the specific challenges facing menopausal women [22]. Women working in cleaning have carried immense responsibility for the wellbeing of others, as have women in care, who have often been confronted with dying and death to an unprecedented degree [40]. Women whose older age, ethnicity, and/or health renders them more vulnerable to COVID-19 have additional concerns, as have those who combine work with their caring responsibilities for clinically-vulnerable family members or friends. Emerging research increasingly suggests that decreasing levels of oestrogen at menopause causes women at menopause to be at particular risk from the COVID-19 virus [44, 45]. Menopausal women caring for elderly or disabled family or friends have been particularly limited in the work they can undertake because most want to avoid not only contracting the virus themselves, but also transmitting it to those for whom they are caring [46].

## 6 Conclusion

Through this review, we have proposed some ways in which menopausal experiences of women in casual work may be distinct from those of women more securely employed in similarly low-paid, low status or manual jobs. Given the relative dearth of research focusing specifically upon the menopausal experiences of women in casual work [21–23], we acknowledge that our assertions are based upon limited evidence. Within the evidence we have reviewed, the terminologies, definitions, and understandings of what “menopause” or “menopausal women” are so heterogenous and imprecise as to preclude any direct or quantifiable comparisons between specific datasets. We selected the methodology of narrative review, then, in order to foreground the broadest qualitative themes, as opposed to establishing the causal links of a realist synthesis, and as opposed to providing the replicable evidence of a systematic review. By foregrounding and describing the predominant themes within the literature it reviews, a narrative review seeks simply to inform the need for future research, and to stimulate debate.

One of our main findings was, however, that work was often not the main challenge which women face [21–23]. In light of this, we wish to caution against the appropriation of this review for any economic or political agenda. Certainly, we have found that casual work may not directly cause ill-health and may indeed provide psychological benefits to some menopausal women. However, we have also found that casual work itself is both a cause and a symptom of poverty, social exclusion and intersectionally gendered social injustice.

Instead, we hope that this review might assist in highlighting the limits of the workplace menopause policies upon which contemporary UK research is largely focused on informing [1–4]. As such menopausal women in casual jobs will likely not benefit from the recommendations, innovations or protections of the ‘menopause at work’ policies introduced by organisations or trade unions [2, 47]. Given, however, that this review has identified particular levels of psychological resilience amongst casual workers, it should not be assumed that casual workers have the same needs as the employees upon whose experiences existing policy research is based [1–4]. Further research specifically investigating the menopausal experiences of casual workers in cleaning and care settings during the COVID-19 pandemic is especially needed.

## Supplementary Material

Supplementary Appendix

## Footnotes

1 Most of the included quantitative studies did not specify their definition of “menopause” or “menopausal women”, and it appears that the majority applied no objective clinical criteria to determining the reproductive status of participants. All of the qualitative studies appeared to use these terms purely in the popular or colloquial sense in which participants described their own “menopause”, as is the longstanding tradition within medical research exploring the lived experiences of women [9, 17]. In the absence of consensus from the quantitative studies, and given our use of the narrative review methodology which inherently privileges the themes derived from lived experience, we use the term “menopausal” in this subjective, flexible, and popular sense.
