# Supplementary material for: Menopausal symptoms and work: a narrative review of women’s experiences in casual, informal, or precarious jobs
Source: Maturitas. Author manuscript; Available in PMC 2021 Aug 1. (PMC7611109; doi:10.1016/j.maturitas.2021.05.007)
Supplement: Supplementary Appendix [file EMS128089-supplement-Supplementary_Appendix.docx]

# **Appendix**

## **Figure 1 Search Protocol**


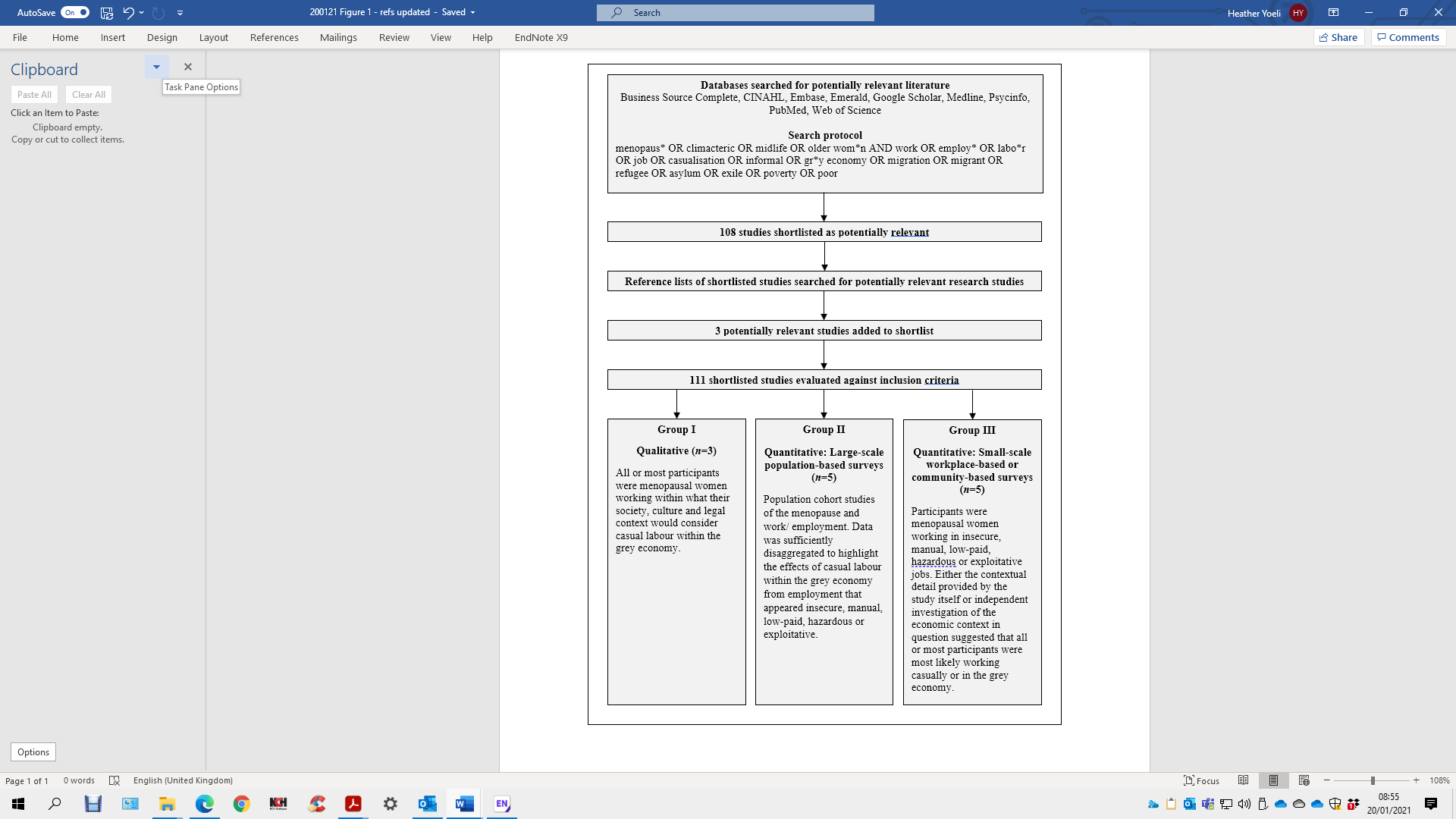


## **Figure 2 Studies selected for review**

### **Group I Qualitative (*n*=3)**

| **AUTHOR** |  | **AIM** | **SETTING** | **PARTICIPANTS** | **METHODS** | **FINDINGS** |
| --- | --- | --- | --- | --- | --- | --- |
| Audet et al. [21] | 2017 | The objective of this qualitative study is to examine the interaction between socioeconomic status (SES) and body weight in order to understand underprivileged women’s increased vulnerability to chronic diseases after menopause (p.1412) | Sherbrooke, Quebec, Canada. Part of the SOMET study, funded by Canadian Institute for Health Research, exploring women’s health behaviours | 20 post-menopausal French-speaking women of BMI <25 whose socioeconomic status was judged as placing them within an "economic underclass" (p.1414) Women with long-term medical conditions were excluded from the study, though women with long-term mental health challenges were included. | Interviews, analysed according to theories of Bourdieu | All participants were either unemployed or precariously-employed in unskilled jobs that offered little economic security. Women felt that their health was not a priority and that they had little control over their health; they therefore were not motivated to lose weight. Work, working conditions and poverty formed only part of their overall life challenges and disadvantage, and they felt little control over their working lives. |
| Delanoë et al. [22] | 2012 | To qualitatively compare the menopause experiences of Tunisian women in Tunisia, Tunisian women in France and French women in France | Study co-authored by researchers in Paris, France and Tunis, Tunisia | 75 women 45-70 years old in both countries: 35 Tunisian women in Tunisia (17 in urban areas, and 18 in rural areas); 20 Tunisian women in France …. and 20 French women in France (FF). We classified as working class the Tunisian women who were illiterate or who had not gone beyond primary school. We considered as middle-class women in Tunisia with any high school education, in France those who had graduated high school, and in both countries, those who had ... more education or ... or worked in low to middle-level management (p.402) | Interviews. These "were recorded, transcribed, and [when conducted in Arabic] translated into French. We studied the interviews by applying propositional content analysis (Bardin, 2007), which included semantic, contextual, attitudinal, and categorical analyses" (p.403) | Work was reported as a challenge only by the "peasant" women of rural Tunisian origin doing heavy manual work; these women "felt ill and complained of reduced physical strength, fatigue, stomach aches and headaches, pain in the back, pelvis, buttocks, knees, leg, feet, and hands" (p.304) and to an extent also hot flushes accompanied by fever and sexual challenges. These women felt that they were entering old age and had become socially "useless". Authors found that middle-class Tunisian and French women experienced either "menopause as loss of aesthetic and social value" or a "tranquil and unstigmatised menopause" within which challenges emotional/ interpersonal rather than physical/ social. |
| Im & Meleis [23] | 2001 | To describe how first-generation Korean immigrant women in the USA describe their work, and to understand how they relate their work to their "symptoms experienced during midlife" (p.83) | Korean immigrant community within an un-named US city | 119 women aged 40-60 "who engaged in low-income/low-status wage work outside their own houses" (p.85) and were literate in Korean. Of these, 26% were formally employed (most often as healthcare assistants, childcare and checkout workers), 32% worked for family businesses, and the remainder worked informally as babysitters, cleaners and in other manual jobs. 21 women were interviewed. | Mixed methods: questionnaires followed by interviews. Interviewing was guided by grounded theory principles of purposive sampling, unstructured questioning and inductive analysis. Findings were established through triangulation of quantitative and qualitative findings. | Women discussed work in four ways: (1) women should be women, (2) women should make career sacrifices to others, (3) domestic work should be women's work and (4) women should work like ants. Some women found that the physical symptoms of menopause (most commonly fatigue and pain) made it difficult or impossible for them do manual work, and changed jobs or took HRT to address this. Whenever women struggled with depression and other psychological symptoms, women responded by working harder - they saw work as beneficial to their mental health. Authors discuss gendered stresses of immigrant experiences. |

### **Group II Quantitative: Large-scale population-based surveys (*n*=5)**

| **AUTHOR** |  |  | **AIM** | **SAMPLE** | **WORK CATEGORIES** | **OTHER VARIABLES** | **FINDINGS** |
| --- | --- | --- | --- | --- | --- | --- | --- |
| Cassou et al. [24] | 1997 | France | To determine whether working conditions are a risk factor for premature menopause | Occupational health records from "a large random sample of gainfully employed [sic] French women born 1938 (n = 1743) and 1943 (n = 1959)" (p.165) | blue-collar/clerks/executives: detailed anaylsis of physical/manual work duties, exposure to occupational hazards | marital status, education, parity, oral contraceptive, breastfeeding, smoking | (1) No association between working conditions and premature menopause; (2) Lower levels of education is associated with an increased risk of premature menopause |
| Evandrou et al. [25] | 2021 | UK | To identify how patterns of female employment changed between aged 50 and 55, to explore how this was associated with menopausal symptoms and socioeconomic status | Questionnaires and interviews with 3019 female participants from UK National Child Development Study Wave 8 and Wave 9. However, excluded all self-employed women. | (1) Socioeconomic status of job (eg professional, managerial, clerical, technical, manual, routine etc); (2) Job satisfaction; (3) Job security; (4) Whether job interferes with family life; (5) Whether (pre-menopause) health or disabilities interfere with work | marital status, employment status and job security of partner, education, health, disability, HRT use | (1) Women in manual/routine jobs tended to experience more menopausal symptoms  (2) Women in higher SES roles reduced their working hours; women in lower SES roles left the workplace  (3) The relationship between menopause symptoms and work is mediated not only by SES but by how work affects personal relationships and family life |
| Lawlor et al. [26] | 2003 | UK | "To assess the association of indicators of adverse socio-economic position from across the life course with age at menopause" (p.1078) | Part of the British Women’s Heart and Health Study:"3513 women aged 60–79 years... Women … for whom a biological age at menopause could not be calculated [eg due to hysterectomy, HRT use during perimenopause] were excluded from this study." (p.1078) | "the longest held occupation of the participant and her spouse" (p.1081) | "longest held occupation of the participant’s father during her childhood; whether the house that the participant lived in for the longest time as a child had a bathroom and … hot water supply; whether as a child the participant had shared or had her own bedroom; whether the childhood household had access to a car; the age at which the participant completed full-time education ... the participant’s current housing tenure; current car access and pension arrangements..." (p.1082) | (1) Adverse socioeconomic circumstances during childhood are associated with younger age at menopause; (2) Socioeconomic status and experiences during childhood have more impact upon menopause age than adult socioeconomic status |
| Lim et al. [27] | 2019 | South Korea | “whether, in the Korean population, the risk of premature ovarian insufficiency (POI) and early menopause varies with a woman’s socioeconomic status, evaluated in relation to income, education,  and occupation” (p.22) | “cross-sectional, population-based study involv[ing] 31,508 women aged >19 years registered in the  Korea National Health and Nutrition Examination Survey (KNHANES) (2007–2016)” (p.22) | “Occupation was categorised as  unemployed, non-manual (white-collar workers), or manual (blue-collar workers). The unemployed women were classified as ‘no job’, students, and homemakers. Managers, experts and related workers, and office workers were classified as non-manual workers. Workers in sales  and services, agriculture, forestry, fishery, engineering, assembling,  technical work, and manual labour were classified as manual workers” (p.23) | marital status, income, education, BMI, smoking, alcohol use | “the prevalence of both POI and early menopause was statistically significantly associated with lower socioeconomic status, such as those with lower household income and lower educational levels” (p.24) Whereas educational background and overall family income correlated with age at menopause, the nature of a woman’s work did not. |
| Mishra & Kuh [28] | 2006 | UK | "relationship between perceived change in QOL [quality of life] and menopausal transition status, socio-economic circumstances, lifestyle factors, and life stress." (p.93) | Part of the MRC 1946 longitudinal birth cohort study. 1025 women (aged 48-54 at time of data collection) completed all relevant questionnaires. *See also Ballard et al. (2001)* | changes in work life, work stress, manual/non-manual social class | marital/ relationship status, family stresses, financial hardship, number of children, BMI, physical activity, smoking | (1) All factors related to work showed no direct association with changes to QOL at menopause; (2) Other socioeconomic variables were more strongly correlated - in particular, higher levels of education appear to protect against decreased QOL |

### **Group III Quantitative: Small-scale workplace-based or community-based surveys (*n*=5)**

| **AUTHOR** |  |  | **AIM** | **SAMPLE** | **WORK CATEGORIES** | **OTHER VARIABLES** | **WORK FINDINGS** | **OTHER FINDINGS** |
| --- | --- | --- | --- | --- | --- | --- | --- | --- |
| Alquaiz et al. [29] | 2013 | Saudi Arabia | "To determine the relationship between sociodemographic, reproductive and lifestyle factors and the severity of menopausal symptoms among Saudi women" (p.1) | 490 women aged 40+ attending outpatient appointments or accompanying relatives to outpatient appointments at Riyadh hospital | working/housewife | education, marital status, living conditions, eating and exercise habits, BMI, parity | Housewives experienced more menopausal symptoms than women who work | Severity of symptoms associated with less affluent housing conditions and lower levels of health-promoting behaviours |
| Coronado et al. [30] | 2020 | Spain | “To assess the impact of confinement due to the coronavirus (Covid-19) pandemic on health-related quality of life (HRQoL) and resilience in peri- and postmenopausal women” (p.4) | 2430 peri-menopausal or post-menopausal Spanish-speaking respondents aged 40-70 to online survey carried out during May 2020 | not working before pandemic/working from home/working outside the home/not working due to pandemic | education, relationship status, caring responsibilities, access to outdoor space, physical activity, sexual activity | (1) Women who had lost jobs due to the pandemic had significantly reduced HRQoL  (2) Work was not otherwise a significant determinant of HRQoL or resilience | (1) Living alone, intense caring responsibilities and being unable to exercise or access outside space was associated with descreased wellbeing.  (2) Women who engaged in frequent physical or sexual activity were more resilient |
| Huseth-Zosel et al. [31] | 2014 | China | To explore the impact of "employment status, occupation and education" (p.98) on menopausal symptoms experienced by urban Chinese women | Part of a larger epidemiological study: 296 women who no longer menstruated | white collar/blue collar/not working | educated/uneducated, married/unmarried | Blue-collar workers had more menopausal symptoms | Less educated women had more menopausal symptoms |
| Loutfy et al. [32] | 2006 | Egypt | "To determine symptoms, perceptions and practices after natural menopause by women aged 50–59 years" (p.S93) | 70 women aged 50-60 in Alexandria, Egypt. Stratified sampling to include all socioeconomic groups. More than 90% of participants illiterate and not working. | urban working/urban nonworking/rural working/rural non-working/squatter working/squatter non-working | education, work status, marriage, income sufficiency | Employment and education are equally predictive of knowledge about menopause | Women regarded menopause as a personal matter than should not be discussed with others and should not be treated medically |
| Ogurlu et al. [33] | 2011 | Turkey | "To evaluate the variation of climacteric symptoms among working and nonworking postmenopausal Turkish women" (p.204) | Randomly-sampled patients of health centre: 66 in menopause, 66 not. Women aged 40-60. Excluded women with hysterectomy or taking HRT | working/ nonworking | income greater than/less than daily living expenses, marital status, BMI | Nonworking women suffered significantly more headaches, hot flushes and urinary symptoms than working women | Significant differences between educational levels of working and nonworking women. Authors suggest this causes differences in hygiene practice to account for urinary symptoms |
